# The Effect of Trier Social Stress Test (TSST) on Item and Associative Recognition of Words and Pictures in Healthy Participants

**DOI:** 10.3389/fpsyg.2016.00507

**Published:** 2016-04-12

**Authors:** Jonathan Guez, Rotem Saar-Ashkenazy, Eldad Keha, Chen Tiferet-Dweck

**Affiliations:** ^1^Department of Psychology, Achva Academic CollegeBeer-Tuvia, Israel; ^2^Ben-Gurion University of the Negev Faculty of Health Sciences, Beer-Sheva Mental Health CenterBeer-Sheva, Israel; ^3^Department of Cognitive Neuroscience and Zlotowski Center for Neuroscience, Ben-Gurion University of the NegevBeer-Sheva, Israel; ^4^Department of Psychology and the School of Social-Work, Ashkelon Academic CollegeAshkelon, Israel

**Keywords:** associative memory, item-recognition, associative-recognition, anxiety, TSST, induce stress, associative-deficit

## Abstract

Psychological stress, induced by the Trier Social Stress Test (TSST), has repeatedly been shown to alter memory performance. Although factors influencing memory performance such as stimulus nature (verbal/pictorial) and emotional valence have been extensively studied, results whether stress impairs or improves memory are still inconsistent. This study aimed at exploring the effect of TSST on item versus associative memory for neutral, verbal, and pictorial stimuli. 48 healthy subjects were recruited, 24 participants were randomly assigned to the TSST group and the remaining 24 participants were assigned to the control group. Stress reactivity was measured by psychological (subjective state anxiety ratings) and physiological (Galvanic skin response recording) measurements. Subjects performed an item-association memory task for both stimulus types (words, pictures) simultaneously, before, and after the stress/non-stress manipulation. The results showed that memory recognition for pictorial stimuli was higher than for verbal stimuli. Memory for both words and pictures was impaired following TSST; while the source for this impairment was specific to associative recognition in pictures, a more general deficit was observed for verbal material, as expressed in decreased recognition for both items and associations following TSST. Response latency analysis indicated that the TSST manipulation decreased response time but at the cost of memory accuracy. We conclude that stress does not uniformly affect memory; rather it interacts with the task’s cognitive load and stimulus type. Applying the current study results to patients diagnosed with disorders associated with traumatic stress, our findings in healthy subjects under acute stress provide further support for our assertion that patients’ impaired memory originates in poor recollection processing following depletion of attentional resources.

## Introduction

### Stress and Episodic Memory

Stress and its associated hormonal, cognitive, and behavioral cascades have been extensively studied. One common procedure for stress induction under laboratory conditions is the Trier Social Stress Test (TSST), which induces stress in a psychological (and not physiological) way. In this procedure, which consists of an anticipation period and a test period, subjects are asked to deliver a free speech and perform a mental arithmetic task in front of a committee. The committee does not give any feedback (verbal or non-verbal) to the subject and only takes occasional notes. Considerable changes in the concentration of adrenocorticotropic hormone (ACTH), cortisol, growth hormone (GH), and prolactin, as well as significant increases in heart rate, have been reported following the TSST (Kirschbaum et al., 1993). Therefore, this procedure has become an acceptable method of inducing stress in psychological experiments.

Several studies have explored the effect of TSST-induced stress (Kirschbaum et al., 1993) on episodic memory, however, conflicting results are reported (reviewed in Wolf, 2009). There are studies that show that TSST impair memory for emotional information across different stimulus modalities; For example, Kuhlmann et al. (2005) have shown that TSST impaired memory of negative, but not neutral, words. TSST was also reported to impair memory for emotional faces (Li et al., 2013). In the study of Quesada et al. (2012), children exposed to the TSST showed lower performance in a delayed memory retrieval test for pictures (i.e., committed more errors). There was a trend towards a more pronounced effect with negative items; however, the results did not reach significance. Other studies have shown that TSST impair memory for neutral, rather than emotional, information; For example, TSST was reported to impair memory for neutral faces (Li et al., 2013). Other studies have reported that TSST impaired memory for neutral, but not emotional, words (Smeets et al., 2006; Stawski et al., 2009; Dreifus et al., 2014). While these studies have shown that TSST can impair memory, there are reports that TSST has improved memory performance; There are studies that showed that TSST improved memory of negative words (Jelici et al., 2004; Luethi et al., 2008) and emotional (versus non-emotional) pictures (Cornelisse et al., 2011). Preuss and Wolf (2009) have reported that TSST improved memory performance for neutral, but not for emotionally arousing, pictures. In contrast to all studies who showed TSST has either positive or negative influence on memory performance, there are studies who reported that TSST did not have any impact on memory for either neutral or emotional pictures (Wolf et al., 2001; Zwissler et al., 2011).

### Stress and Memory Processes

Understanding the effect of psychosocial stress (e.g., TSST) on associative recognition for neutral, non-autobiographical information carries practical and theoretical implications, since according to the dual-process theory, episodic memory is based on two independent contributing processes, familiarity, and recollection. While familiarity is associated with a vague experience of remembering and is relatively automatic in nature, recollection involves executive functioning, and is associated with a clear sense of remembering (see Jacoby, 1991; Yonelinas, 2002). Task analysis of paradigms that tested for the difference in familiarity/recollection processes using item-association memory tasks suggests that whereas item recognition may rely on both familiarity and recollection, associative recognition predominantly relies on recollection processes (Old and Naveh-Benjamin, 2008).

To date, only few studies have tested the effect of psychosocial stress on neutral, non-autobiographical memory (for items and for associations) in healthy as well as in clinical populations. It is also not clear whether TSST has similar effects across different types of stimuli (e.g., words vs. pictures). Wiemers et al. (2013) tested the effect of stress on associative memory and showed that TSST enhanced memory consolidation of information acquired during stress. Specifically, the authors showed that an arousing situation makes an associated object more memorable: central details of a stressful episode are better remembered than those of a non-stressful episode. The authors concluded that the information remembered from a stressful episode is dependent on the strength of the association between the stressor and the material to be remembered. Takahashi et al. (2004) tested associative memory between names and faces, before and after TSST. While no behavioral differences in performance were evident before and after the TSST, a significant negative correlation between cortisol elevation and memory performance was found, supporting the hypothesis that acute stress impairs memory. Notwithstanding, differences (or their absence) in memory performance following TSST were not addressed in the context of the dual-process theory and require further investigation. Understanding the mechanism underlying memory performance in acute stress conditions can shed light on the way stress differentially affects recollection and familiarity processes as manifested in item versus associative recognition.

### Stress and Reaction Time

Memory accuracy and reaction time (RT) have been discussed as interacting (Kahana and Loftus, 1999), and stress has been shown to alter RT in various cognitive tasks. For example, there are reports that stress eliminates increases in RT under incompatible (compared to compatible) conditions in a flanker-interference task (Sato et al., 2012). Others have showed that stress improves dual-task efficiency by shortening RT in short (as compared to long) stimulus onset asynchronies (Beste et al., 2013). In contrast, stress has been reported to increase RT in an n-back task (a forced-choice task in which subjects are required to monitor series of briefly presented stimuli and decide if the currently presented stimuli is the same as the one presented *n* trials backwards), yet this effect was significant only at the beginning of the task and disappeared following the first block (Schoofs et al., 2008). Notwithstanding, reports regarding the impact of stress on RT in episodic memory tasks are still lacking. Interestingly, chronic stress patients demonstrate increased RTs as compared to healthy controls while performing memory recognition tasks (Saar-Ashkenazy et al., 2014). These (and similar) results were recently found associated with changes in brain anatomical connectivity in chronic stress patients (Saar-Ashkenazy et al., 2016) and raise the importance of further studying the interaction between stress and RT in episodic memory tasks. The question whether individuals under acute (rather than chronic) stress show slower or faster RTs has not been addressed in the literature of episodic memory thus require further investigation.

### Stress and Memory Accuracy – Evidence from Patients

Recent studies conducted on populations with disorders associated with traumatic stress (Old and Naveh-Benjamin, 2008; Guez et al., 2011, 2013; Saar-Ashkenazy et al., 2014) raised the hypothesis that associative memory impairments occur because of impaired recollection, i.e., when an encoded unit is retrieved with partial/without its contextual information (Old and Naveh-Benjamin, 2008). This impairment in binding individual sensory features into stable objects can in turn lead to incomplete/false memories (Brewin, 2011), as seen in populations with disorders associated with traumatic stress. Focusing on the distinction between familiarity and recollection under stress conditions may be a way to uncover the mechanisms underlying memory impairments in clinical populations. However, research in patient populations is often retrospective in its nature, including patients with co-morbid disorders (Danckwerts and Leathem, 2003) who have been symptomatic for years with wide variations in both time elapsed since the trauma and medication effects; all of these factors may cause changes in cognition that are not strictly related to stress symptomatology (Savić et al., 2005). These methodological issues make it difficult to draw firm conclusions regarding the effect of stress on cognition in general, and specifically, on associative memory (Danckwerts and Leathem, 2003; Isaac et al., 2006). Therefore, supporting evidence from analog studies conducted with healthy participants is required.

### The Current Study

In the current study we aimed to expand the understanding of the effect of psychosocial stress on familiarity versus recollection-based processes in healthy participants by employing an item-association memory paradigm, which allows distinguishing between item recognition (which is largely familiarity-based) and associative recognition (which is based on recollection processes). Differential memory performance for verbal versus pictorial stimuli has been reported in populations with stress-related disorders (Guez et al., 2013; Saar-Ashkenazy et al., 2014), with a trend exists towards a more pronounced impairment for verbal stimuli (Bustamante et al., 2001; Kleim and Ehlers, 2008). We therefore tested the effect of TSST on both types of stimuli (words and pictures). Forty-eight participants were recruited and randomly assigned to the TSST or control group. Overall, participants performed four blocks of the item-association paradigm, two preceding and two following the manipulation. In each block, participants viewed a different learning list that was followed by an item recognition test and an association recognition test. RT was recorded. In accordance with our previous results in patients (Guez et al., 2011, 2013) as well as healthy participants (Guez et al., 2015), we hypothesized that under conditions which prompt acute psychosocial stress, retrieval of verbal and pictorial stimuli is based more on familiarity than on recollection. Thus, the TSST manipulation was expected to impair associative recognition, whereas item recognition should remain preserved.

## Materials and Methods

### Participants

Participants in the current study were 48 psychology students (*M*_(years)_ = 22.95, *SD* = 2.23 and *M*_(years)_ = 23.41, *SD* = 2.41; with three and four male participants in the control and experimental group, respectively) from Achva Academic College that were rewarded for their participation with course credit, an acceptable procedure in a first-year introductory psychology academic course. Twenty-four participants were randomly assigned to the TSST group and the remaining 24 participants were assigned to the control group. All participants reported being in good health and without a formal diagnosis of learning disabilities. Exclusion criteria included current sensory/motor disorders (participants with corrected vision were able to participate in the study) and past/current psychiatric or neurological disorders (as was confirmed by the participants in a self-report). The study was approved by the local institutional review board of Achva Academic College. All participants gave their written informed consent for study participation.

### Experimental Design

Four independent variables were used: *Test* (a within-subject variable, item/associative recognition), *Time* (a within-subject variable, pre/post manipulation), *Stimuli* (a within-subject variable, words/pictures), and *Group* (a between-subject factor, TSST/control). The dependent variables were *response latency* (in milliseconds) and *memory accuracy* that was calculated as the percentage of hits minus the percentage of false-alarms. A hit occurs when the participant correctly identifies a target test-item as a target and a false-alarm occurs when a distracter/lure is, erroneously, identified by the participant as a target. With this measure, chance level performance (guessing) yields a score of 0.00 and perfect performance yields a score of 1.00.

### Memory Paradigm

In the current study we used a procedure applied previously by the authors (Guez et al., 2011, 2013, 2015; Saar-Ashkenazy et al., 2014, 2016) to test for specific impairments in associative memory. This procedure included a learning phase and two memory tests: item recognition and associative recognition.

#### Stimuli

The process of stimulus pool creation, from which stimuli were drawn to the current study, involved several steps. Firstly, a set of 280 high-frequency common Hebrew nouns (based on the norms in Rubinsten et al., 2005) was chosen. From this set, we created 120 pairs of unrelated (semantically or phonologically) words. The additional 40 words were kept to serve as distracters in the item-recognition test lists. Finally, for each word, a matched line-drawing picture was created. Stimuli were adjusted so that no significant differences between the number of black pixels in the items of the word set and the items of the picture set were evident (*M*_(pixels)_ = 2485, *SD* = 789 for words and *M*_(pixels)_ = 2371, *SD* = 1323 for pictures; *t*(279) = 1.203; *p* = 0.217).

**FIGURE 1:**
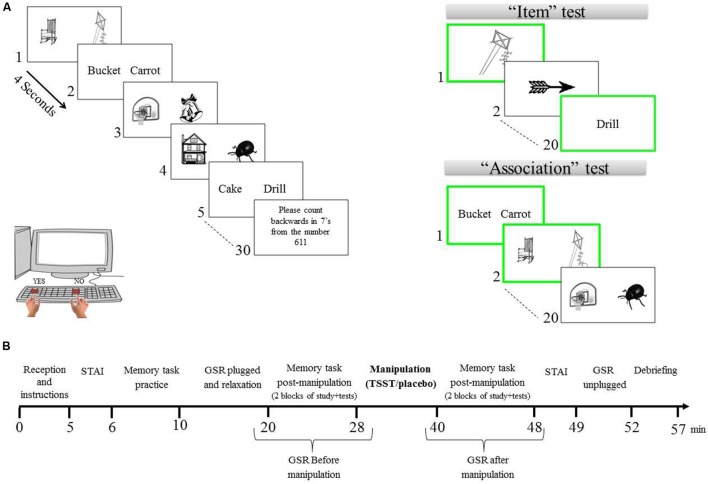
**(A)** Schematic representation of the procedure and memory task. Words and picture pairs were presented within each list and were counterbalance between subjects (study list was presented to half of the subjects and its mirror lists [pictorial stimuli were replaced by their words representation and vise versa] for the other). All stimuli were presented in white against a black background. **(B)** Schematic time line of the study procedure. Stress manipulation was checked using STAI and GSR measurements before and after the TSST. Before the TSST a memory task was employed. In this task, subjects were asked to complete two blocks. A different list was presented in each block followed by item and association tests. Two additional blocks were employed after the TSST.

For the learning phase, four lists of 30 pairs were created from the set of 120 pairs. Each list contained 15 word-pairs and 15 picture-pairs. Then, a complementary mirror set of learning lists was created by substituting each word pair with its counterpart picture pair, and vice versa. Thus, two versions for each list were created. In each learning phase, participants were asked to study a list of pairs presented on a 15” computer monitor. Learning was intentional: participants were instructed to learn both the individual stimuli and the pairs and were informed that following this phase, they will be asked to perform a recognition test for individual items and for associations between stimuli. The learning phase was followed by a 30-second distraction task (counting backward in sevens from a randomly selected number) to prevent rehearsal between the learning and memory test. Each learning list was followed by item and associative recognition tests. The order of the stimuli within lists was randomized. The order of the tests (i.e., item test and association test) was counter-balanced within participants and the order of the learning lists was counter-balanced across participants (see **Figure 1**).

#### Item Recognition Test

The list for the item-recognition test included 20 stimuli, 10 words, and 10 pictures. Five of each stimulus type were targets (i.e., appeared in the learning phase) and five distracters (i.e., new stimuli that had not appeared in the learning phase, but shared similar characteristics as the target stimuli), mixed randomly. Participants were informed that the list included targets and distracters, and were instructed to respond to each stimulus with a designated “yes” key for targets and a “no” response key for distracters.

#### Associative Recognition Test

The list for the associative-recognition test included 20 pairs, 10 intact pairs (targets) and 10 distracters (i.e., rearranged pairs that contained the same items from the learning list that were now recombined and presented as novel pairs), mixed randomly. As in the item recognition test, participants were instructed to respond by pressing the “yes” key for targets, and the “no” key for the distracters (rearranged pairs). Stimuli that were used in the item test were not used in the associative test and vice-versa.

### TSST Manipulation

A modified version of the original TSST procedure (Kirschbaum et al., 1993) was applied. Participants underwent a mock job interview simulation in front of one, unfamiliar, female interviewer. Participants were given two minutes to prepare a short oral speech about their personal traits qualifying them for their “dream” job position and were then asked by the interviewer to start their presentation while being recorded via video-camera. During the presentation the interviewer took notes occasionally but did not give any feedback. After five minutes of presentation the subjects were asked to count backwards in steps of 17 from 2574. When subjects made a mistake, the interviewer asked them to start over. After five minutes the TSST ended and subjects were instructed to wait in the room for the experimenter to proceed to the second part of the experiment. In the placebo–TSST (i.e., control condition), after two minutes of preparation, the subjects stood alone in the room and talked out loud on a given subject (their last vacation). After five minutes, participants completed an easy mathematical assignment were they counted forwards in steps of 15 from 0. No interviewer was present during this test, and subjects were not recorded via a video-camera. The duration of both the TSST/placebo-TSST manipulation was 12 minutes.

### Manipulation Check

To assess anxiety levels we used the Spielberger’s State-Trait Anxiety Inventory (STAI); a self-report questionnaire that measures state and trait anxiety. The STAI questionnaire contains 20 items and each item is rated on a 4-point Likert scale (range from ‘1’ = not at all to ‘4’ = very much). The total scores of this measure are obtained by summing the values assigned to each item, and range from a minimum of 20 to a maximum of 80, with higher scores indicating more severe anxiety symptoms (Spielberger et al., 1983). Participants were given standardized instructions before completing the STAI questionnaire. Prior to the experiment, all participants from both groups filled the Trait Anxiety Inventory (TAI). In addition, participants filled the State Anxiety Inventory (SAI) twice; before and after the stress manipulation. While the STAI serves as a self-report, subjective, measure for anxiety, Galvanic skin response (GSR) was used as an additional physiological measure for assessing anxiety levels. GSR is a method of capturing the autonomic nerve response as a parameter of the sweat gland function (i.e., measuring the electrical resistance of the skin). As stress level increases, changes in the electrical resistance of the skin are detected by GSR sensors. Since GSR varies with moisture levels, skin conductance is used as an indication of psychological or physiological arousal. GSR recording was performed using the NeXus-10 mobile and wireless recording device (which is manufactured by Mind Media, for more information see http://www.mindmedia.nl/english/nexus10.php). Skin conductance was recorded at a rate of 32 samples/second using a two fingers NeXus-10-SC/GSR sensor. We attached one sensor to the middle finger and the other sensor to the ring finger of the left hand. For each subject, a mean score of SC was calculated twice; before and after the manipulation (see **Figure 1** for schematic representation of study lists and procedure).

## Results

### Manipulation Check

#### State-Trait Anxiety Inventory Scores

No significant difference in the Spielberger trait Anxiety Inventory (TAI; Spielberger et al., 1983) was found between groups before the manipulation [*M*_(control)_ = 40.50 *SD* = 9.00, *M*_(TSST)_ = 38.20, *SD* = 6.98, *t*(46) = 0.984, *p* = 0.329]. To assess the effectiveness of the TSST/placebo-TSST manipulation we tested the differences in the SAI before and after the manipulation. The results of the two-way mixed-design analysis of variance (ANOVA) [*Group* (TSST, control) X *Time* (pre/post manipulation phase)] with repeated-measures on the second factor (*Time*) indicated significant main effect for *Group* [*F*(1,46) = 8.463, *p* = 0.005, $\eta_{p}^{2}$ = 0.15]; a significant main effect for *Time* [*F*(1,46) = 38.619, *p* = 0.000, $\eta_{p}^{2}$ = 0.45] and more importantly, the interaction of these two variables was significant [*F*(1,46) = 15.508, *p* < 0.001, $\eta_{p}^{2}$ = 0.25]. Planned comparisons analysis showed that no differences in state-anxiety levels were evident between the groups at the pre-manipulation phase [*M*_(control)_ = 33.00, *SD* = 5.18, *M*_(TSST)_ = 32.95, *SD* = 6.51, *F*(1,46) = 0.000, *p* = 0.980, $\eta_{p}^{2}$ = 0.00] but a significant difference in state-anxiety levels was observed between groups at the post-manipulation phase [*M*_(control)_ = 36.62 *SD* = 10.58, *M*_(TSST)_ = 49.12, *SD* = 12.66, *F*(1,46) = 13.764, *p* < 0.001, $\eta_{p}^{2}$ = 0.23; see also **Table 1**].

**Table 1:** State-Trait Anxiety Inventory (STAI), Galvanic skin response (GSR), and memory performance in the pre and post manipulation phases for the negative emotional arousal and control groups.

		Placebo-TSST group				TSST group			
		Pre-manipulation		Post-manipulation		Pre-manipulation		Post-manipulation	
		*M*	*SD*	*M*	*SD*	*M*	*SD*	*M*	*SD*
TAI		40.50	9.00			38.20	6.98		
SAI		32.66	5.37	36.87	11.07	32.95	6.51	49.12	12.66
GSR		4.94	4.08	5.07	4.45	4.80	3.63	8.27	9.27
Words items	Hit	0.75	0.20	0.67	0.26	0.79	0.14	0.63	0.20
recognition	FA	0.12	0.17	0.12	0.16	0.15	0.16	0.26	0.19
Words association	Hit	0.74	0.21	0.71	0.26	0.76	0.23	0.60	0.21
recognition	FA	0.17	0.14	0.14	0.19	0.15	0.19	0.18	0.17
Pictures items	Hit	0.88	0.12	0.82	0.21	0.91	0.14	0.84	0.15
recognition	FA	0.06	0.09	0.03	0.06	0.09	0.11	0.07	0.11
Pictures association	Hit	0.87	0.17	0.87	0.19	0.90	0.12	0.79	0.17
recognition	FA	0.20	0.22	0.16	0.16	0.17	0.15	0.18	0.15
Response latency (ms) Words item test		1534	360	1400	480	1622	485	1253	360
Response latency (ms) Words associative test		1795	385	1875	410	1985	523	1792	500
Response latency (ms) Pictures item test		1406	467	1425	501	1451	431	1278	435
Response latency (ms) Pictures associative test		1902	443	1810	485	1879	478	1800	477

*FA, False Alarms*.

#### Galvanic Skin Response (GSR)

Galvanic Skin Response (GSR) measures before and after TSST/ placebo-TSST manipulation in each group are presented in **Table 1**. One participant from the control group and one participant from the experimental group (i.e., TSST) were excluded from the analysis due to technical problems.

The results of the two-way mixed-design analysis of variance (ANOVA) [*Group* (TSST, control) ×*Time* (pre/post manipulation)] indicated a significant main effect for *Time* [*F*(1,44) = 4.425, *p* = 0.041, $\eta_{p}^{2}$ = 0.09]; with no significant main effect for *Group* [*F*(1,44) = 1.053, *p* = .310, $\eta_{p}^{2}$ = 0.02]. More importantly, the interaction of these two variables was close to significance [*F*(1,44) = 3.817, *p* = 0.057, $\eta_{p}^{2}$ = 0.08]. Planned comparisons analysis showed that while no differences in skin conductance levels were evident between the pre/post manipulation phase in the control group [*M*_(pre)_ = 4.94, *SD* = 4.08, *M*_(post)_ = 5.07, *SD* = 4.45, *F*(1,44) = 0.011, *p* = 0.916, $\eta_{p}^{2}$ = 0.00], a significant difference in skin conductance levels was observed in the experimental (i.e., TSST) group [*M*_(pre)_ = 4.80, *SD* = 3.63, *M*_(post)_ = 8.27, *SD* = 9.27, *F*(1,44) = 8.231, *p* = 0.006, $\eta_{p}^{2}$ = 0.16)].

### Memory Performance

Overall, memory recognition for pictorial stimuli was significantly higher than for verbal stimuli [*F*(1,46) = 71.617, *p* < 0.001, $\eta_{p}^{2}$ = 0.608].

#### Memory Performance for Verbal Stimuli

**FIGURE 2:**
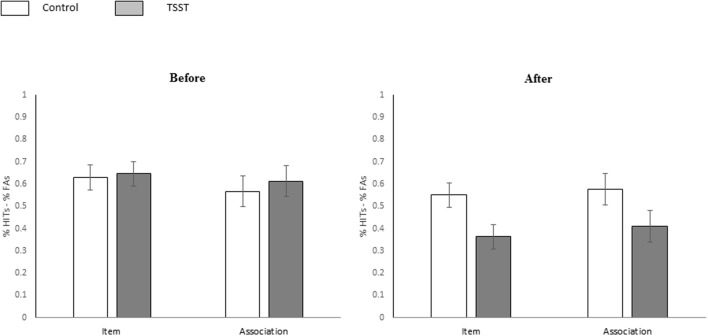
**Memory performance for verbal stimuli.** Memory performance (%Hit–%FAs) in the item and the associative recognition tests for word stimuli in the control and TSST groups, before and after the stress manipulation. Error bars represent the standard error of the mean.

The mean proportions of hits and false alarms for each *Test* (item and associative recognition), and experimental phase (i.e., *Time*, pre-post manipulation) for each group are shown in **Table 1**. To specifically address the hypothesis tested in this experiment we employed a three-way mixed-design ANOVA [*Group* (TSST, control) × *Time* (pre/post manipulation) × *Test* (item and associative recognition)] with repeated measures on the last two variables (*Time* and *Test*) and the dependent variable as *Memory accuracy* (percentage of Hits minus the percentage False-Alarms). The results indicated a significant main effect for Time [*F*(1,46) = 16.61, *p* < 0.001, $\eta_{p}^{2}$ = 0.26; *M*_(pre)_ = 0.613 *SD* = 0.26, *M*_(post)_ = 0.475 *SD* = 0.27), i.e., memory accuracy before the manipulation was higher than after the manipulation. Additionally, a significant two-way interaction between *Time* and *Group* was found [*F*(1,46) = 9.20, *p* = 0.003, $\eta_{p}^{2}$ = 0.17]. Further planned analysis on this interaction effect yielded no difference in memory accuracy between groups before the manipulation [*F*(1,46) = 0.166, *p* = 0.685; *M*_(control_*_)_* = 0.597 *SD* = 0.264, *M*_(TSST)_ = 0.629, *SD* = 0.274], but a significant difference in memory accuracy between groups after the manipulation [*F*(1,46) = 4.84, *p* = 0.033, $\eta_{p}^{2}$ = 0.10; *M*_(control)_ = 0.562, *SD* = 0.264, *M*_(TSST)_ = 0.387, *SD* = 0.274], indicating that participants in the TSST group showed lower performance in memory accuracy after the manipulation as compared with control subjects for both item and associative recognition (see **Figure 2**). No significant main effects for *Group* and *Test* were found [*F*(1,46) = 1.04, *p* = 0.312, $\eta_{p}^{2}$ = 0.02 and *F*(1,46) = 0.02, *p* = 0.872, $\eta_{p}^{2}$ = 0.00, respectively], and no other significant interactions (i.e., between *Test* × *Group* and between *Time* ×*Test*) were found [*F*(1,46) = 0.17, *p* = 0.676, $\eta_{p}^{2}$ = 0.00 and *F*(1,46) = 2.32, *p* = 0.134, $\eta_{p}^{2}$ = 0.05, respectively]. The three way interaction was not significant [*F*(1,46) = 0.00, *p* = 0.970, $\eta_{p}^{2}$ = 0.00].

#### Memory Performance for Pictorial Stimuli

**FIGURE 3:**
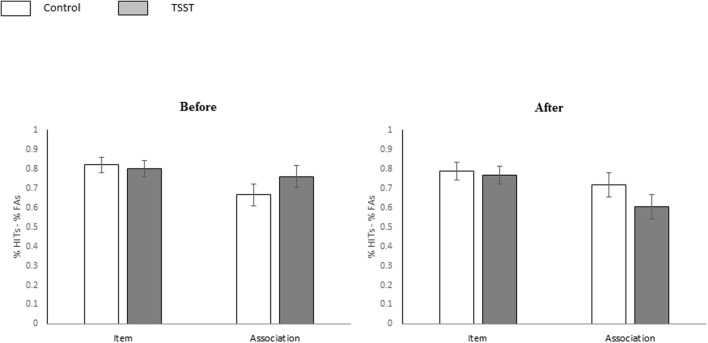
**Memory performance for pictorial stimuli.** Memory performance (%Hit–%FAs) in the item and the associative recognition tests for pictorial stimuli in the control and TSST groups, before and after the manipulation. Error bars represent the standard error of the mean.

The mean proportions of hits and false alarms for each *Test* (item and associative recognition), and experimental phase (i.e., *Time*, pre/post manipulation) for each group are shown in **Table 1**. To specifically address the hypothesis tested in this experiment we employed a three-way mixed-design ANOVA [*Group* (TSST, control) × *Time* (pre/post manipulation) × *Test* (item and associative recognition)] with repeated measures on the last two factors (*Time* and *Test*) and the dependent variable as *Memory accuracy*. The results indicated a significant main effect for *Test* [*F*(1,46) = 18.39, *p* < 0.001, $\eta_{p}^{2}$ = 0.28], with higher performance in the item recognition test as compared with the associative recognition test [*M*_(item)_ = 0.794, *SD* = 0.138, *M*_(associations)_ = 0.679 *SD* = 0.207]. A significant three-way interaction was found between *Time × Test × Group* [*F*(1,46) = 4.24, *p* = 0.003, $\eta_{p}^{2}$ = 0.08]. Further planned analysis on this interaction effect yielded no significant two-way interaction between *Group* and *Time* for item recognition accuracy [*F*(1,46) = 0.003, *p* = 0.955] but a significant interaction for associative recognition accuracy [*F*(1,46) = 4.10, *p* = 0.048, $\eta_{p}^{2}$ = 0.08]. This interaction indicates that while no difference in associative recognition was evident before or after the manipulation in the control group [*F* = 0.669, *p* = 0.417; *M*_(pre)_ = 0.666 *SD* = 0.27, *M*_(post)_ = 0.716, *SD* = 0.29], a significant decrease in associative recognition was observed after the manipulation in the TSST group [*F*(1,46) = 4.18, *p* = 0.046, $\eta_{p}^{2}$ = 0.08; *M*_(pre)_ = 0.729, *SD* = 0.27, *M*_(post)_ = 0.604, *SD* = 0.29; see **Figure 3**]. No significant main effects for *Group* and *Time* were found [*F*(1,46) = 0.15, *p* = 0.691, $\eta_{p}^{2}$ = 0.00 and *F*(1,46) = 1.10, *p* = 0.298, $\eta_{p}^{2}$ = 0.02, respectively], and no other significant interactions [i.e., between *Time* ×*Group*, *Test* ×*Group*, and between *Time* ×*Test* were observed [*F*(1,46) = 1.67, *p* = 0.203, $\eta_{p}^{2}$ = 0.03, *F*(1,46) = 0.01, *p* = 0.908, $\eta_{p}^{2}$ = 0.00, and *F*(1,46) = 0.00, *p* = 0.960, $\eta_{p}^{2}$ = 0.00, respectively].

### Retrieval Latency

For each experimental phase we averaged the latency in each test (in milliseconds). The mean latencies across trials and participants for each condition are shown in **Table 1**.

#### Retrieval Latency for Verbal Stimuli

A three-way mixed-design ANOVA [*Group* (TSST, control) ×*Time* (pre/post manipulation) ×*Test* (item and associative recognition)] with repeated measures on the last two factors (*Time* and *Test*) and the dependent variable as *Response latency* was applied. The results showed significant main effects for *Time* and *Test* [*F*(1,46) = 16.70, *p* < 0.001, $\eta_{p}^{2}$ = 0.26; *F*(1,46) = 73.23, *p* < 0.001, $\eta_{p}^{2}$ = 0.61, respectively] and a significant interaction for *Time* and *Group* [*F*(1,46) = 11.35, *p* = 0.001, $\eta_{p}^{2}$ = 0.19]. *Post hoc* comparisons indicated that while no difference was observed in the control group before and after the manipulation (*F* < 1), response latency was faster in the TSST group following the manipulation [*F*(1,46) = 27.79, *p* < 0.001, $\eta_{p}^{2}$ = 0.37]. The interaction between *Time* and *Test* was also significant [*F*(1,46) = 7.34, *p* = 0.009, $\eta_{p}^{2}$ = 0.14]. *Post hoc* comparisons indicated that while no difference in response latency was observed in the associative recognition task between the pre and post manipulation phases [*F*(1,46) = 1.22, *p* = 0.275, $\eta_{p}^{2}$ = 0.02], in the item recognition test, faster responses were observed following the post manipulation phase as compared to the pre manipulation phase [*F*(1,46) = 22.63, *p* < 0.001, $\eta_{p}^{2}$ = 0.32, see **Table 1**]. The three way interaction did not reach significance (*F* < 1).

#### Retrieval Latency for Pictorial Stimuli

A three-way mixed-design ANOVA [*Group* (TSST, control) × *Time* (pre/post manipulation) × *Test* (item and associative recognition)] with repeated measures on the last two factors (*Time* and *Test*) and the dependent variable as *Response latency* was applied. The results showed significant main effects for *Time* and *Test* [*F*(1,46) = 4.22, *p* = 0.045, $\eta_{p}^{2}$ = 0.08; *F*(1,46) = 102.73, *p* < 0.001, $\eta_{p}^{2}$ = 0.69, respectively]. While the interaction between *Time* and *Group* was not significant [*F*(1,46) = 1.24, *p* = 0.27], specific pair-comparisons revealed similar results pattern as for verbal material, i.e., no difference in response latency was observed in the control group before and after the manipulation (*F* < 1), but a significant decrease in response latency was evident in the TSST group following the manipulation as compared with the pre manipulation phase [*F*(1,46) = 5.02, *p* = 0.029, $\eta_{p}^{2}$ = 0.10]. The effect of *Group* and the two-way interactions (between *Group* and *Test* and between *Time* and *Test)* were not significant (*F* < 1). The three way interaction was not significant [*F*(1,46) = 1.36, *p* = 0.248, $\eta_{p}^{2}$ = 0.03].

## Discussion

In the current study we tested the effect of psychosocial stress (i.e., TSST) on memory recognition for individual stimuli (items) versus associations between stimuli (for both verbal and pictorial stimuli). Confirmation of induced stress was performed using psychological and physiological measurements, both converged and showed a change towards higher stress levels following the TSST manipulation. Overall, memory recognition for pictorial stimuli was higher than for verbal stimuli. Memory for both words and pictures was impaired following TSST; while the source for this impairment was specific to associative recognition in pictures, a more general deficit was observed for verbal material, as expressed in decreased recognition for both items and associations following TSST. Response latency analysis indicated that the TSST manipulation facilitated response speed but at the cost of memory accuracy. This pattern was stronger for verbal stimuli which also exhibited a greater memory deficit.

The findings of the current study are in line with previous studies reporting memory impairment for neutral information following TSST (e.g., Stawski et al., 2009; Dreifus et al., 2014) and other manipulations that tested the effect of induced arousal on memory binding (Nashiro and Mather, 2011; Guez et al., 2015). While there are studies that have reported no group differences in memory accuracy between the stress and the non-stress group (Wolf et al., 2001; Takahashi et al., 2004), these studies reported a negative correlation between high cortisol levels following TSST and poorer memory accuracy. The lack of group differences in the presence of a negative correlation between cortisol and memory can be attributed to a possible power problem, as these samples were relatively small and in the case of Takahashi et al., (2004) were even further divided to high/low responders, resulting in an even smaller sample size for each group.

Notwithstanding, the results of the current study are in contrast to findings that have shown that TSST improved memory for neutral information (Preuss and Wolf, 2009). In this study, the authors employed the TSST manipulation after the learning phase and tested for delayed memory of neutral, positive or negative pictures. Thus, their study tested the effect of stress on the retention phase after encoding and after 24 h while in the current study we tested the effect of stress on both the encoding and the retrieval phases. Other studies, such as that of Zwissler et al. (2011) did not find a significant effect of TSST on memory performance, yet this might be the result of the chosen statistical analysis, which was collapsed over neutral and positive pictures. Examining the impact of TSST separately for neutral and positive pictures reveals a similar pattern to the current study results, with approximately ~8% decrease in memory accuracy of neutral stimuli; however, this analysis was not shown. Overall, it seems that factors such as memory paradigm (free recall versus recognition tests, immediate or delayed), stimulus modality (visual or other) and nature (words, pictures, faces or other) as well as the timing of the manipulation (before, during or after the encoding phase) have an important role in determining memory performance and accuracy, and should be addressed more carefully in future studies.

The different effect of TSST on verbal versus pictorial memory as observed in the current study remains complex; while TSST specifically impaired associative recognition in pictures, a more general deficit was evident for verbal material, as expressed in decreased recognition for both items and associations. This pattern of results can be attributed to an interaction between stress and cognitive demands such that stress enhances cognitive demands, and thus cognitive load (Chajut and Algom, 2003; Sato et al., 2012), and in turn leads to an impaired ability of participants to exert control over the recruitment of selective attention and related brain regions (mainly prefrontal, see related neuroimaging studies McDermott et al., 1999; Habib et al., 2003; Babiloni et al., 2006), resulting in lower memory performance. Since item recognition is considered to rely on both familiarity and recollection, whereas associative recognition dominantly relies on recollection processes (Old and Naveh-Benjamin, 2008), it could be claimed that under high cognitive load (i.e., as induced by stress), memory retrieval shifts towards familiarity-based processes, which are faster and more prone to errors (see also Guez et al., 2015). This view is supported also by the finding that the TSST group showed significantly faster RTs compared to the control group in the verbal task, and consistent with this interpretation, there was a specific deficit for associative recognition for pictures. Although a similar RT pattern was observed in pictures, it was weaker (effect size = 0.1 as compared with 0.37 in the verbal task). An alternative/complementary hypothesis would be that the pictorial task is less cognitively demanding in general and thus more immune to stress conditions. This view is supported by higher memory accuracy in the pictorial task in general. While these hypotheses converge, future studies are warranted to empirically test them under lab-conditions in order to determine which fits the findings best.

Incomplete/false memories as a result of an impaired binding process (i.e., binding individual features into cohesive objects) are frequent and were reported in disorders associated with traumatic stress (Brewin, 2011). Posttraumatic stress disorder (PTSD) is a mental disorder that may develop after a traumatic life event and is characterized by symptoms such as re-experiencing the traumatic event, avoidance of situations associated with it, negative mood and cognition and hyper-arousal (DSM-5; American Psychiatric Association [APA], 2013). One common tendency of PTSD patients is overgeneralization from traumatic cues to unrelated neutral ones (Ehlers and Clark, 2000), resultant in binding together negative and neutral stimuli, and wrong associations between neutral stimuli (Guez et al., 2011; Saar-Ashkenazy et al., 2014). Impaired associative memory and increased bias towards false-memory have been reported in PTSD (Bremner et al., 2000; Zoellner et al., 2000; Golier et al., 2002; Guez et al., 2011; Saar-Ashkenazy et al., 2014) and acute-stress disorder (ASD) patients (Guez et al., 2013). Applied to the mentioned findings in patients, the results of the current study support the hypothesis of impaired recollection, i.e., when an encoded unit is retrieved with partial/without its contextual information (Old and Naveh-Benjamin, 2008), resulting in associative memory impairments. Relating to the RT results of the current study, it seems that while chronic stress is associated with slower performance (Saar-Ashkenazy et al., 2014), possibly due to changes in brain connectivity (Saar-Ashkenazy et al., 2016), acute stress is associated with shorter RT, but at the cost of accuracy. These findings highlight the importance of further studying the interaction between acute and chronic stress to impaired memory and RT and can lead in turn to new insights and interventions in PTSD and related disorders.

To the best of our knowledge this study is the first to attempt to evaluate the reciprocal relationship between stress, representation format (word vs. picture), and memory type (item versus associative). In contrast to previous studies that explored the item/associative memory for either verbal or pictorial stimuli, our paradigm allowed us to evaluate the effect of stress on item/associative memory on both types of stimuli simultaneously under strict control (including constant number of pixels). Since same item and associative concepts were presented both in verbal and pictorial forms, content priority of the word/picture (i.e. the meaning of the concept) is minimized.

Still, several limitations of the current study must be acknowledged. Firstly, whether or not the current study results can be generalized to additional populations (e.g., males, older populations etc.) remains an open question, as our sample consisted mainly of young women, which have been reported to show a stronger negative effect of psychosocial stress on memory as compared to men (Kudielka et al., 1998; Wolf et al., 1998). Secondly, the design used in the current study precludes reaching definitive conclusions on whether the effect of TSST on memory is due to impaired encoding, or retrieval, or both. Thus, further replications using the current paradigm or similar paradigms are required in order to answer these questions.

To summarize, we suggest that acute stress increases cognitive load, resulting in memory impairment in healthy participants. This effect is more pronounced in verbal material which is more demanding in its nature as compared to pictorial material. Applying our findings to patients, we suggest that the fragmented trauma processing seen in PTSD may be related in part to an altered cognitive processing as seen in the current study. This hypothesis is in line with both therapeutic and intervention approaches that focus on the integration of fragmented memories into a cohesive episode in PTSD and other disorders associated with traumatic stress.

## Author Contributions

Conceived and designed the experiments: JG, EK, RS-A, CT-D. Performed the experiments: EK, JG. Analyzed the data: JG, RS-A. Contributed reagents/materials/analysis tools: CT-D. Wrote the paper: RS-A, JG, CT-D.

## Conflict of Interest Statement

The authors declare that the research was conducted in the absence of any commercial or financial relationships that could be construed as a potential conflict of interest.
